# Regulation of *Ubx* Expression by Epigenetic Enhancer Silencing in Response to Ubx Levels and Genetic Variation

**DOI:** 10.1371/journal.pgen.1000633

**Published:** 2009-09-04

**Authors:** Michael A. Crickmore, Vikram Ranade, Richard S. Mann

**Affiliations:** 1 Department of Biological Sciences, Columbia University, New York, New York, United States of America; 2 Department of Genetics and Development, Columbia University, New York, New York, United States of America; 3 Department of Biochemistry and Molecular Biophysics, Columbia University, New York, New York, United States of America; Princeton University, Howard Hughes Medical Institute, United States of America

## Abstract

For gene products that must be present in cells at defined concentrations, expression levels must be tightly controlled to ensure robustness against environmental, genetic, and developmental noise. By studying the regulation of the concentration-sensitive *Drosophila melanogaster* Hox gene *Ultrabithorax* (*Ubx*), we found that *Ubx* enhancer activities respond to both increases in Ubx levels and genetic background. Large, transient increases in Ubx levels are capable of silencing all enhancer input into *Ubx* transcription, resulting in the complete silencing of this gene. Small increases in Ubx levels, brought about by duplications of the *Ubx* locus, cause sporadic silencing of subsets of *Ubx* enhancers. *Ubx* enhancer silencing can also be induced by outcrossing laboratory stocks to *D. melanogaster* strains established from wild flies from around the world. These results suggest that enhancer activities are not rigidly determined, but instead are sensitive to genetic background. Together, these findings suggest that enhancer silencing may be used to maintain gene product levels within the correct range in response to natural genetic variation.

## Author Summary

Gene expression is generally governed by *cis*-regulatory elements, also called enhancers. For genes whose expression levels must be tightly controlled, enhancer activities must be tightly regulated. In this work, we show that enhancers that control the expression of the Hox gene *Ultrabithorax* (*Ubx*) in *Drosophila* are regulated by a negative autoregulatory feedback mechanism. Negative autoregulation can be triggered by less than a two-fold increase in *Ubx* levels or by varying the genetic background. Together, these data reveal that enhancer activities are not always hardwired, but instead may be sensitive to genetic and environmental variation and, in some cases, to the amount of gene product they regulate. The finding that enhancers are sensitive to genetic background suggests that the regulation of gene expression is more plastic than previously thought and has important implications for how transcription is controlled *in vivo*.

## Introduction

The transcriptional control of gene expression in eukaryotes is governed by *cis*-regulatory elements, also known as enhancers, that integrate cell-type and temporal information by binding combinations of transcription factors. Genes that exhibit complex expression patterns are typically controlled by multiple *cis*-regulatory elements, some of which have overlapping, partially redundant activities [1],[2],[3],[4]. Current estimates suggest that from 10 to 80% of the non-coding DNA of higher eukaryotes is devoted to gene regulation [5],[6],[7], raising the question of how all of this regulatory information is integrated to generate accurate and stereotyped patterns of gene expression in space and time. A third dimension of gene regulation is quantity, which is especially relevant for genes that must be expressed within a narrow range of levels. One possible solution is that enhancers are precisely tuned to generate the appropriate level of transcription that is required in each cell. However, the precision that this type of mechanism demands seems difficult to achieve and especially vulnerable to genetic, environmental, and developmental noise. An alternative solution is that feedback or other regulatory mechanisms exist that modulate enhancer activities in response to the levels of gene product. Although feedback autoregulation is a well-known motif in transcriptional networks [8], mechanisms that might be used to tune expression levels are not well understood. This problem is particularly challenging for genes that have multiple, partially redundant regulatory inputs.

**Figure 1:**
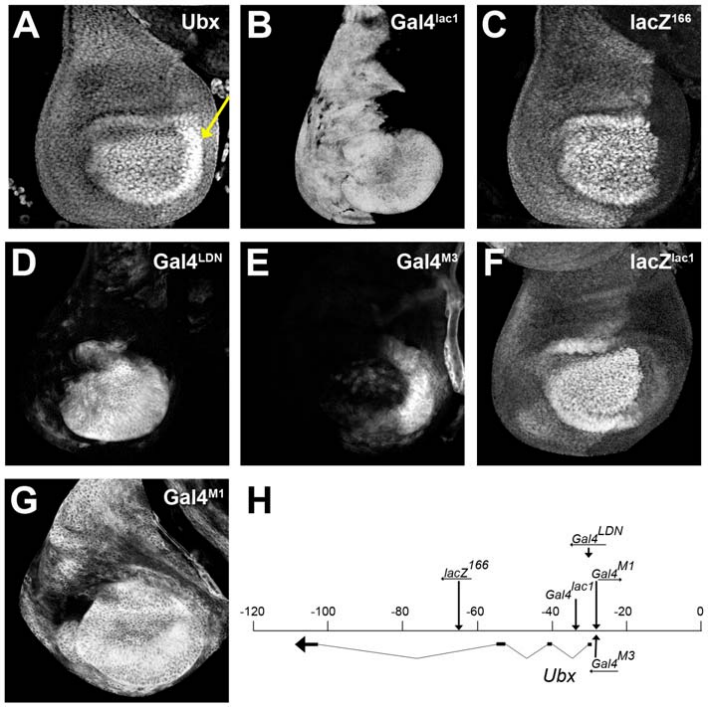
*Ubx* enhancer traps. (A) Haltere disc stained for Ubx protein. Note the higher levels in the center of the disc and in the P compartment (arrow). (B–G) Patterns of *Ubx* enhancer trap expression in wild type haltere discs. The Gal4 inserts were monitored using a *UAS-GFP* transgene. (H) Map of the *Ubx* locus showing the location of the *Ubx* enhancer traps as described previously [28],[29],[30].

We have begun to study this problem in the fruit fly, *Drosophila melanogaster*, by analyzing the mechanisms that control the expression of the Hox gene *Ultrabithorax* (*Ubx*) in the haltere–a dorsal appendage on the third thoracic segment (T3) that helps the fly balance during flight [9]. Although Ubx protein is detected in all cells of the developing haltere imaginal disc, its pattern of expression is not uniform [10] (Figure 1A). Subsets of the complex regulatory input into the *Ubx* locus can be monitored by examining the expression patterns of *Ubx* enhancer traps, which exhibit different, overlapping subsets of the *Ubx* expression pattern (Figure 1). *Ubx-Gal4^lac1^*, for example, (monitored with *UAS-GFP*) is expressed uniformly throughout the anterior (A) compartment of the haltere disc, but only in the distal portion of the posterior (P) compartment (Figure 1B). In contrast, *Ubx-Gal4^LDN^* is expressed in distal regions (in both the A and P compartments) but is not expressed proximally (Figure 1D).

## Results/Discussion

### *Ubx* negative autoregulation

**Figure 2:**
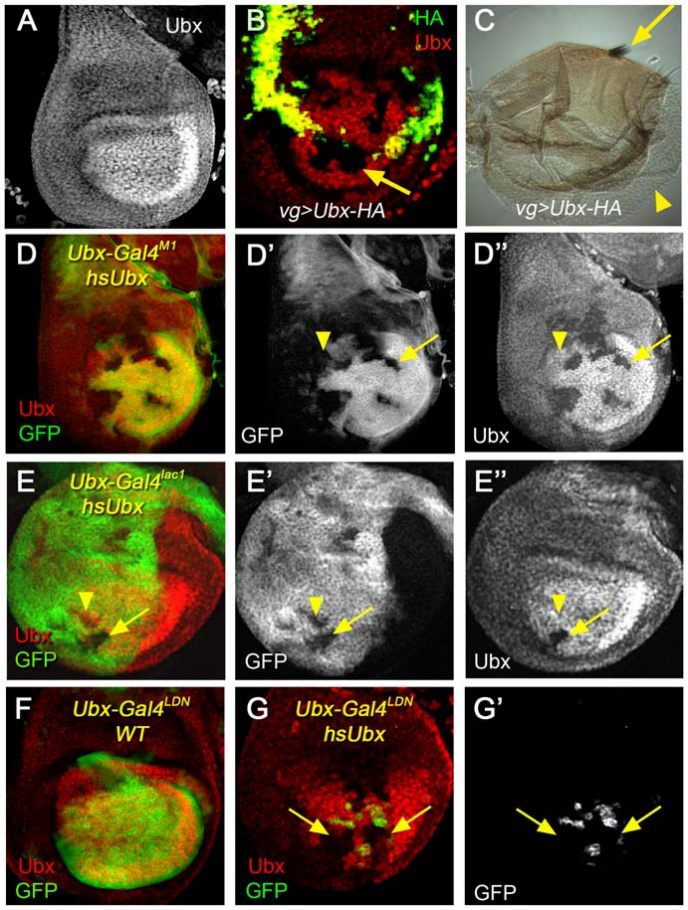
*Ubx* enhancer silencing in response to *hs-Ubx*. (A) Wild type haltere disc stained for Ubx protein. Note the higher levels in the distal region. (B) Haltere disc in which an HA-tagged Ubx protein was expressed via the *vg-Gal4* driver, which is transiently expressed in all haltere cells. The disc was stained for HA (green) and Ubx (red). At this stage, the *vg-Gal4* driver is active along the dorso-ventral boundary (strong green and yellow stain). Groups of cells that do not stain for Ubx (arrow) are observed. (C) Adult haltere from a *vg>Ubx* fly showing a transformation from haltere to wing. Both wing margin (arrow) and wing blade (arrowhead) tissue is observed. (D,E) *Ubx-Gal4^M1^* (D) and *Ubx-Gal4^lac1^* (E) haltere discs that were given a transient pulse of Ubx expression by heat shock during the 2nd instar, stained for GFP (green, to monitor enhancer trap activities) and Ubx (red). Some cells no longer express the enhancer traps and Ubx (arrows). Some cells no longer express the enhancer traps, but still express Ubx (arrowheads). (F) Wild type *Ubx-Gal4^LDN^* haltere disc stained for GFP (green, to monitor the enhancer trap) and Ubx (red). (G) A *Ubx-Gal4^LDN^* haltere disc that was given a transient pulse of Ubx expression by heat shock during the 2nd instar, stained for Ubx (red) and GFP (green, to monitor the enhancer trap). Silencing of both Ubx and the enhancer trap are observed (arrows). Surrounding the Ubx silenced cells, some cells have reduced Ubx levels but still express the enhancer trap.

Somewhat paradoxically, transient ectopic expression of Ubx, induced either by heat shock or Gal4-mediated expression, resulted in *Ubx* loss-of-function transfomations that can be visualized both in the adult (as haltere to wing transformations; [11]) and in 3rd instar haltere imaginal discs (as groups of cells that showed a reduction or complete loss of Ubx protein) [12] (Figure 2). Thus, a transient pulse of high Ubx protein levels can lead to the complete and heritable silencing of all *Ubx* expression, implying that *Ubx* is being silenced by its own gene product.

Transient pulses of ectopic Ubx also resulted in the stable silencing of *Ubx* enhancer traps, including *Ubx-Gal4^lac1^*, *Ubx-Gal4^M1^*, *Ubx-Gal4^LDN^*, and *Ubx-lacZ^166^* (Figure 2 and Table S1). When the absence of Ubx protein was observed, these cells also had no enhancer trap expression (Figure 2). However, in many cases enhancer trap silencing was observed in cells that had normal Ubx protein levels (Figure 2). In these cases we suggest that only the enhancers captured by the enhancer trap were silenced, and that other, partially redundant, enhancers in the *Ubx* locus remained active, resulting in an apparently normal pattern of Ubx expression. We also find, consistent with previous results [12], that the patches of *Ubx*-silenced cells in the haltere are clonal events and that the Polycomb system of epigenetic regulators is required for silencing (Figure S1 and Figure S2).

**Figure 3:**
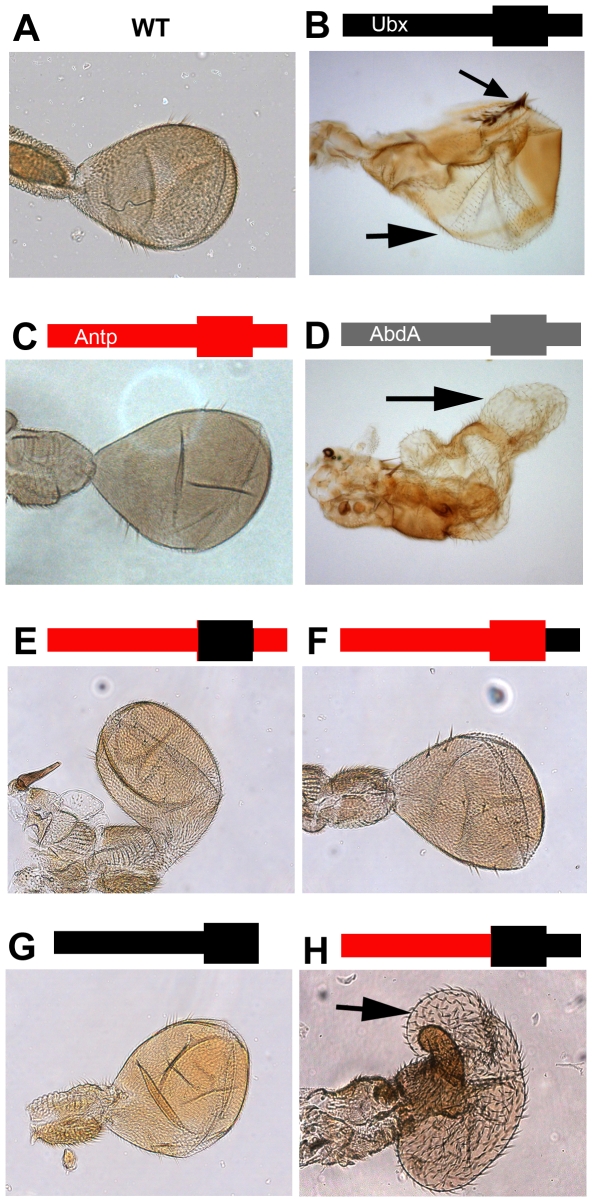
*Ubx* Silencing requires the Ubx homeodomain and C-terminus. (A) Wild type haltere. (B) *vg-Gal4 UAS-Ubx* halteres show haltere to wing transformations due to *Ubx* silencing. (C) *vg-Gal4 UAS-Antp* halteres fail to produce any haltere to wing transformations. (D) *vg-Gal4 UAS-AbdA* halteres show haltere to wing transformations that are indistinguishable from those seen with *UAS-Ubx*. AbdA and Ubx have very similar homeodomains and also share the UbdA motif in the C-terminus, consistent with these domains playing a critical role in silencing. (E) *vg-Gal4 UAS-AUA* (Antp N-terminus, Ubx homeodomain, Antp C-terminus) halteres show no transformation to wing in 8/10 samples and mild transformations in 2/10 samples. (F) *vg-Gal4 UAS-AAU* (Antp N-terminus, Antp homeodomain, Ubx C-terminus) halteres show no haltere to wing transformations. (G) vg-Gal4 UAS-UU* (Ubx N-terminus, Ubx homeodomain, deletion of the C-terminus) halteres show no haltere to wing transformations. (H) *vg-Gal4 UAS-AUU* (Antp N-terminus, Ubx homeodomain, Ubx C-terminus) halteres show haltere to wing transformations indistinguishable from those seen with *UAS-Ubx*.

To obtain initial mechanistic insights into *Ubx* autoregulatory silencing, we carried out experiments that suggest it requires specific DNA binding by Ubx. For these experiments, we monitored the ability of chimeric Hox proteins to induce haltere-to-wing transformations when expressed via the *vg-Gal4* driver. Although the more anterior Hox protein Antennapedia (Antp) was unable to induce *Ubx* silencing, transient overexpression of Antp-Ubx chimeric proteins revealed that the Ubx homeodomain and adjacent C-terminal sequences were both necessary and together sufficient to induce robust *Ubx* silencing (Figure 3). These findings suggest that Ubx protein, and not *Ubx* mRNA, is responsible for the induction of silencing. Further, as both the homeodomain and adjacent sequences are implicated in Ubx specificity and DNA binding [13],[14],[15], these results suggest that Ubx triggers silencing by binding to Ubx-specific *cis*-regulatory elements. Consistently, the Hox protein Abdominal-A (Abd-A), which is very similar to Ubx in both domains, also induced *Ubx* silencing when transiently expressed during haltere development (Figure 3).

### *Ubx* enhancer silencing triggered by additional copies of the *Ubx*+ gene

**Figure 4:**
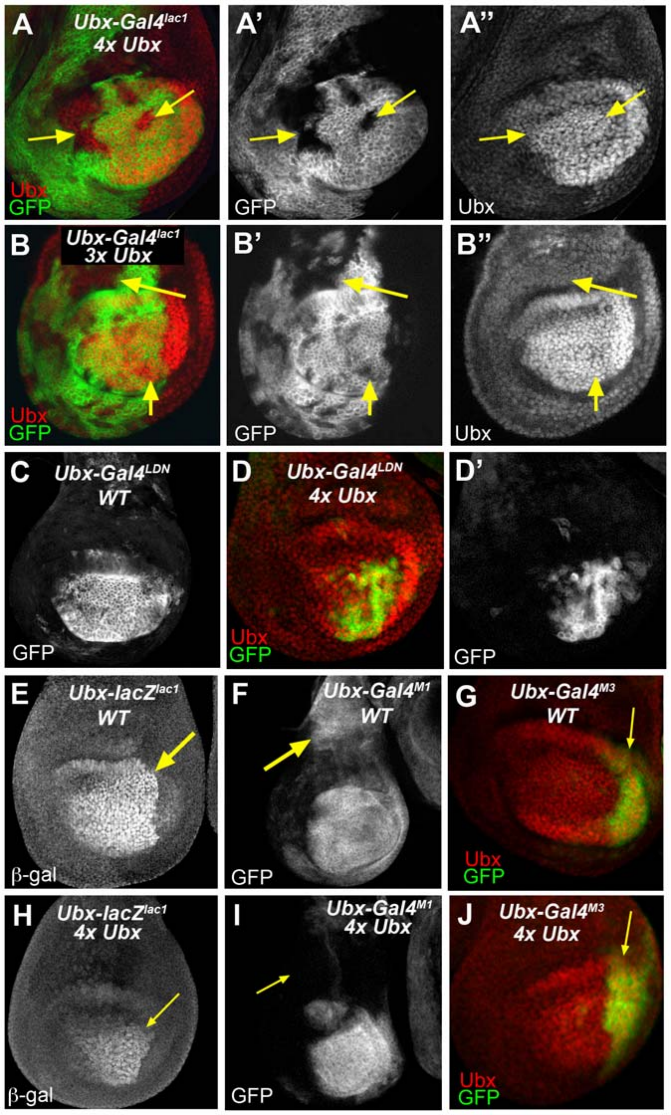
*Ubx* enhnacer trap silencing in response to increasing *Ubx+* dose. (A) *Ubx-Gal4^lac1^* is silenced in groups of cells by 4 copies of the *Ubx+* locus (arrows), but Ubx protein levels are normal. (B) *Ubx-Gal4^lac1^* is silenced in groups of cells by 3 copies of the *Ubx+* locus (arrows), but Ubx protein levels are normal. (C) Wild type haltere expression pattern of *Ubx-Gal4^LDN^*. (D) *Ubx-Gal4^LDN^* is partially silenced by 4 copies of the *Ubx+* locus. (E–G) Wild type haltere expression patterns of *Ubx-lacZ^lac1^* (E), *Ubx-Gal4^M1^* (F), and *Ubx-Gal4^M3^* (G). (H–J) *Ubx-lacZ^lac1^* (H) and *Ubx-Gal4^M1^* (I), but not *Ubx-Gal4^M3^* (J), are partially silenced by 4 copies of *Ubx+*. Note that for *Ubx-lacZ^lac1^* and *Ubx-Gal4^M1^*, silencing does not occur in random clones, but instead is manifest by a loss of expression in proximal regions of the disc (arrows).

We next tested whether more subtle increases in Ubx levels could also induce silencing. For these experiments, we monitored the expression of *Ubx lacZ* or *Gal4* enhancer traps in flies that had extra copies of the wild type *Ubx* locus. *Ubx-Gal4^lac1^* and *Ubx-Gal4^LDN^* were silenced in groups of haltere cells of 3x *Ubx+* and 4x *Ubx+* flies (100% of 4x *Ubx*+ haltere discs had at least one group of silenced cells) (Figure 4A–4D; Table S1). In these haltere discs, probably because the flies had multiple copies of *Ubx+*, the pattern of Ubx protein was invariably wild type (Figure 4A, 4B, 4D). Interestingly, the amount of silencing induced by 4 copies of *Ubx* was significantly decreased when one of these copies encoded a non-functional Ubx protein (the *Ubx^9–22^* allele; data not shown). This result supports the idea that Ubx protein, not *Ubx* mRNA, is the inducer of silencing in response to extra copies of the *Ubx* locus.

*Ubx-Gal4^M1^* and *Ubx-lacZ^lac1^* responded differently to 4x *Ubx+*: instead of being silenced in clones, these enhancer traps were no longer expressed in proximal regions of the haltere disc, but distal expression remained unchanged (Figure 4E, 4F). For *Ubx-lacZ^166^*, the levels were strongly reduced in 4x *Ubx+* flies compared to 2x *Ubx+* flies (Table S1). Note, however, that *Ubx-lacZ^166^* can be completely silenced in clones in response to *hs-Ubx* (Figure S3 and Table S1). Finally, the expression of *Ubx-Gal4^M3^* did not change in the presence of four copies of the *Ubx+* locus (Figure 4G and Table S1). Taken together, these results allow us to make three important conclusions. First, silencing is occurring at the level of *Ubx* enhancers, not entire *Ubx* alleles, because different *Ubx* enhancer traps respond in different ways. Second, silencing can be triggered by the presence of only one or two additional *Ubx+* loci, suggesting that less than doubling *Ubx* levels is sufficient to silence some enhancers. Third, although all *Ubx* enhancers can be silenced by high Ubx levels, lower Ubx levels result in a range of responses that depend on which enhancer trap, and therefore which subset of *Ubx* enhancers, is being monitored. Thus, we conclude that different *Ubx* enhancers are sensitive to different levels of Ubx protein. We also generated flies to monitor two different enhancer trap insertions into the *Ubx* locus (*Ubx-lacZ^166^* and *Ubx-Gal4^lac1^*) at the same time. When silencing was triggered by heat shock-induced *Ubx*, we observed silencing of both enhancer traps, but at different frequencies: *Ubx-Gal4^lac1^* was silenced to a greater extent than *Ubx-lacZ^166^* (Figure S3). This finding provides additional support for the idea that individual enhancer traps, and thus different subsets of *Ubx* enhancers, respond differently to the same increase in Ubx levels.

### Haltere size and Ubx levels are buffered in response to increased *Ubx+* copy number

**Figure 5:**
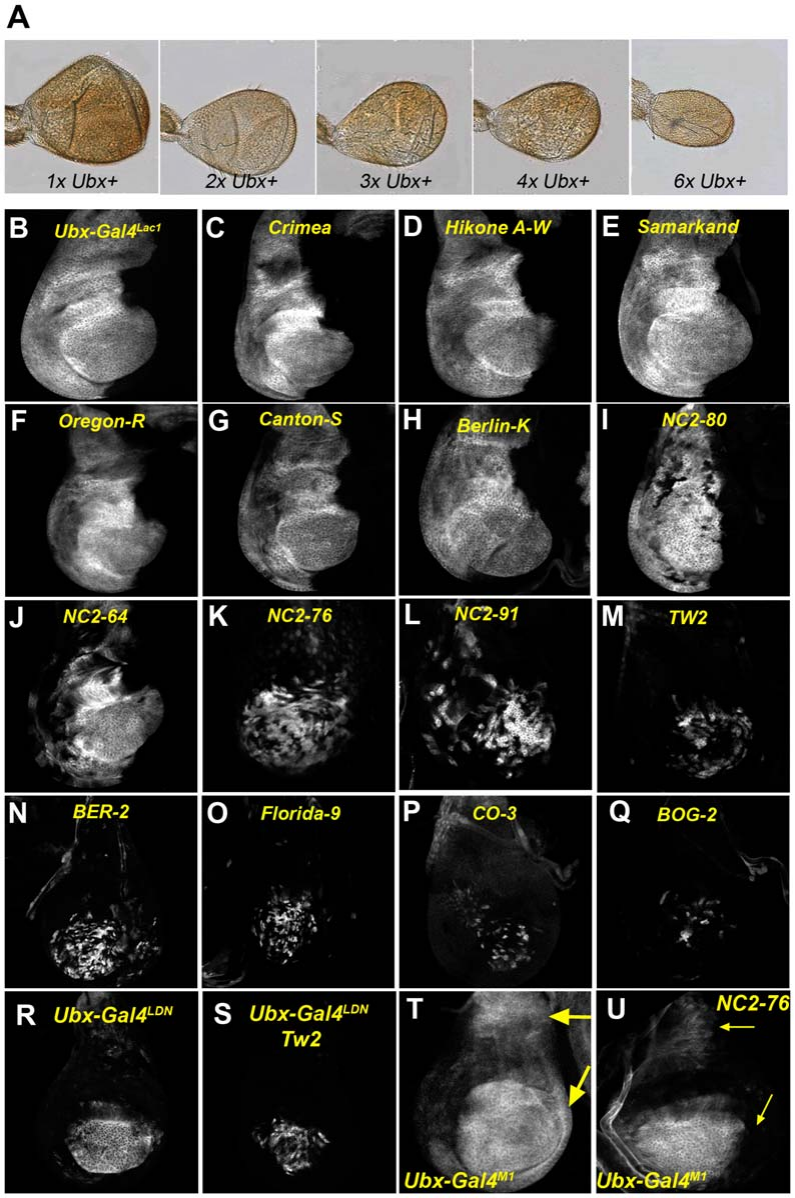
*Ubx* enhancer silencing in response to natural genetic variation. (A) Halteres decrease in size with increasing *Ubx+* copy number. *UbxDf(109)/+*(*1xUbx+*); Wild Type (*2xUbx+*); *Dp(P5)/+*(*3xUbx+*); *Dp(P10)2x/+*(*4xUbx+*); *Dp(P10)2x/+; Dp(P5)/Dp(P5)* (*6xUbx+*). (B–U) All images show haltere discs stained for enhancer trap expression. (B–Q) *Ubx-Gal4^lac1^* driven UAS-GFP reporter expression in the lab stock (B) and outcrossed to various wild type stocks (C–Q). Stocks beginning with “*NC2*” were collected in North Carolina. Other wild type stocks were obtained from the Bloomington Stock Center. See Table S1 and Table S2 for a complete summary of these results. (C–H) Outcrossing to these stocks does not cause *Ubx-Gal4^lac1^* silencing. (I–L) Outcrossing to these wild type stocks causes mild to moderate *Ubx-Gal4^lac1^* silencing. (M–Q) Outcrossing to these wild type stocks causes moderate to strong *Ubx-Gal4^lac1^* silencing. (R,S) *Ubx-Gal4^LDN^* in the lab background (R) and in F1 progeny when crossed to Tw2 (S). Strong clonal silencing is observed. (T,U) *Ubx-Gal4^M1^* in the lab background (T) and in F1 progeny when crossed to NC2-76 (U). Loss of proximal expression (arrows) is observed.

The above results show that epigenetic autoregulatory silencing of *Ubx* enhancers occurs in response to elevated Ubx levels. Interestingly, increasing the dose of *Ubx+* results in smaller halteres [16], but this size change does not scale linearly with the number of *Ubx+* genes. Haltere size is similar to wild type in flies with 3x *Ubx+* or 4x *Ubx+*, while in flies with 6 copies of *Ubx+*, haltere size is greatly reduced (Figure 5A and Figure S4A). These results suggest that haltere size is buffered against increasing doses of the *Ubx+* gene. A similar buffering can be observed when Ubx protein levels are quantified in haltere discs from animals with different numbers of *Ubx+* genes. When one copy of *Ubx* is inactivated (1x *Ubx*+), Ubx protein levels are nearly halved (Figure S4A). However, when the *Ubx+* complement is doubled (4x *Ubx*+) or tripled (6x *Ubx*+) only 39% and 60% increases in Ubx protein levels were detected, respectively (Figure S4A). The less-than-expected increases in Ubx levels seen in Ubx duplications is not because they fail to express wild type levels, as they are sufficient to fully rescue a *Ubx* null mutation, both phenotypically [17],[18] and with respect to Ubx protein levels (data not shown). Together with the results described above, we suggest that the buffering of Ubx levels and haltere size is due, at least in part, to the epigenetic silencing of *Ubx* enhancers in response to higher than normal doses of *Ubx+*.

### *Ubx* enhancer silencing induced by genetic variation

**Figure 6:**
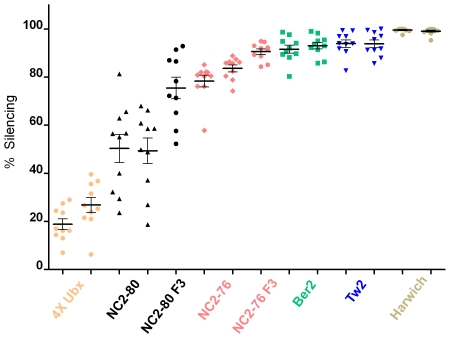
Quantification of silencing. Each point records the % silencing of the *Ubx-Gal4^lac1^* enhancer trap for a single haltere disc. % silencing is defined as the amount of non-stained tissue relative to wild type controls measured in parallel (see Materials and Methods for details). Unless otherwise indicated, all measurements were of haltere discs from F1 animals grown under non-crowded conditions produced by crossing our laboratory *Ubx-Gal4^lac1^* stock to the indicated genetic backgrounds (4× *Ubx*, orange circles; NC2-80, black triangles; NC2-76, pink diamonds; Ber2, green squares; Tw2, blue triangles; Harwich, tan circles). Silencing was measured in two independent sets of crosses, separated in time by two weeks, and are graphed in neighboring columns. The thick black bars correspond to averages and the thinner bars show the standard error of the mean. For each cross, a minimum of 10 haltere discs, from 10 different animals, were scored. An analysis of variance (ANOVA) shows that the differences among the five wild type genotypes (NC2-80, NC2-76, Ber2, Tw2 and Harwich) in % silencing were highly significant (t ratio = 9.4, p = 0.0007) with 83% of the variance among lines, and no differences between replicates. Also graphed is the % silencing measured in 10 independent haltere discs resulting from the continued introgression (F3 generations) of *Ubx-Gal4^lac1^* into the NC2-80 background (black circles) and into the NC2-76 background (pink circles). The average % silencing increased in the F3 generations compared to the F1 generations.

In wild type animals, we hypothesized that enhancer silencing may be used to ensure uniform Ubx levels in response to naturally occurring genetic variation in the *cis*- and *trans-*regulation of *Ubx* expression. We tested this idea by out-crossing our laboratory *Ubx-Gal4^lac1^* flies to 32 *D. melanogaster* strains established from wild populations around the world. In our lab stock, less than 5% of haltere discs showed any evidence of *Ubx-Gal4^lac1^* silencing. However, when outcrossed to wild *D. melanogaster* strains, we frequently observed silencing of *Ubx-Gal4^lac1^* in haltere discs of the F1 generations (Figure 5 and Table S2). Although the frequency of silencing varied between wild stocks, it was consistent for each wild stock in a statistically significant manner (Figure 6). Of the 32 stocks crossed to *Ubx-Gal4^lac1^*, 14 resulted in no detectable silencing in the F1 generation, 6 showed weak silencing in the F1 generation, and 12 showed strong silencing in the F1 generation (Figure 5 and Table S2). Because the amount of silencing can, in some cases, approach 100% (e.g. Tw2 F1), while 4x *Ubx+* resulted in ∼20–30% silencing (Figure 6), we suggest that differences beyond Ubx levels contribute to silencing in these F1 outcrosses. Genetic variation may, for example, result in differences in the levels or activities of the *trans*-regulators of *Ubx*. Silencing was also observed when *Ubx-lacZ^lac1^* and *Ubx-Gal4^LDN^* were outcrossed to wild populations, demonstrating that this effect is not limited to *Ubx-Gal4^lac1^* (Figure 5R–5U and Table S1). Despite the silencing of *Ubx* enhancer traps, the pattern and levels of Ubx protein were similar in the wild stocks, our laboratory stocks, and in their F1 progeny (Figure S4B). We ruled out that the lack of enhancer trap expression in these outcrosses was due to a failure to initiate expression by carrying out a lineage tracing experiment, which demonstrates that *Ubx-Gal4^lac1^* was expressed prior to silencing (see Materials and Methods). We also ruled out that transposon instability (e.g. hybrid dysgenesis [19]) was responsible for the loss of enhancer trap expression using several criteria (see Materials and Methods). Most importantly, silencing occurred at the same frequency when the male or female parent was from the wild (non-laboratory) stock and the amount of enhancer trap DNA, measured by qPCR, was unchanged between the parental and F2 generations. Further, silencing of enhancer traps in other genes, including *Distalless-Gal4*, *homothorax-lacZ*, and *teashirt-lacZ* was not observed by crossing these insertions to the same wild strains (data not shown).

We postulate that silencing induced in these outcrosses may be due to an incompatibility between the *trans*-acting factors (largely derived from the wild stocks) and *cis*-regulatory elements (linked to the monitored *Ubx* locus of the laboratory stock) controlling *Ubx* expression. In support of this idea, when *Ubx-Gal4^lac1^* was further introgressed into weakly or strongly silencing wild stocks, which effectively increases the genetic complement from the wild strain background, an increase in the severity of silencing was observed when compared to the F1 generation (Figure 6 and Figure S5). We also never observed the complete absence of Ubx protein or haltere-to-wing transformations in any of these outcrosses, arguing that only a subset of enhancer inputs into *Ubx* is silenced in response to genetic variation. Consistently, individual enhancer traps responded differently when crossed to the same wild strains (Table S1).

Together, these results demonstrate that *Ubx* enhancer silencing is triggered when Ubx is present at higher than normal levels. When Ubx concentration is especially high (when Ubx is ectopically expressed via Gal4 or heat-shock promoters) all enhancer input into *Ubx* can be silenced, resulting in the complete absence of *Ubx* expression and haltere-to-wing transformations. Although such high levels of Ubx are not physiological, we also find that *Ubx* enhancer silencing can be triggered by additional copies of *Ubx+*, which in principle results in less than double the amount of Ubx protein. In this case, we find that the expression of some *Ubx* enhancer traps is clonally silenced (e.g. *Ubx-Gal4^lac1^*), while the expression of other enhancer traps (e.g. *Ubx-lacZ^166^*) is reduced. Thus, different *Ubx* enhancers are differentially sensitive to negative autoregulation; some are shut off by relatively low Ubx levels, while others require high Ubx levels to be silenced.

### Enhancer silencing and natural genetic variation

Most remarkably, we found that enhancer silencing can occur simply by varying the genetic background. In *Drosophila melanogaster*, due in part to its large population size, the frequency of DNA polymorphisms between individuals in the wild is estimated to be as high as 1 in 100 basepairs [20]. Due to these polymorphisms, we imagine that different strains of *D. melanogaster*, when kept in isolation from each other, may have subtly different ways of regulating *Ubx*. These may be due to strain-specific differences in the *Ubx cis*-regulatory elements, in the *trans* regulators of *Ubx* expression, or both. Consistent with this idea, it is of interest that gene expression levels, when assayed across entire genomes, show a lot of variability in natural populations [21],[22],[23],[24],[25]. Although we find that the final Ubx expression pattern and levels are very similar between lab and wild *D. melanogaster* strains, when two strains are bred together genetic differences may result in fluctuations in the initial Ubx levels. The silencing system described here may function to compensate for these fluctuations and thus ensure that the correct Ubx levels are produced throughout the haltere.

### Plasticity of enhancer activities

In the crosses to wild *D. melanogaster* strains, we found that the expression of genetically marked *Ubx* alleles varied tremendously, depending on the genetic background. Extrapolating from these results suggests that there is a lot of previously undetected variability in enhancer activities at the *Ubx* locus in wild files that would not have been detected using traditional assays. Thus, these results challenge the standard view that a given transcriptional enhancer integrates the same inputs and produces the same outputs, regardless of genetic background. Instead, due to natural genetic variation, the activity of a particular enhancer may vary widely between individuals in wild populations. Additionally, our results show that the activity of an enhancer can even vary among the cells within its expression domain (e.g. the haltere) in a single individual. We suggest that plasticity in enhancer activities is essential to compensate for genetic and perhaps environmental variation. Moreover, given that many genes may have multiple, partially redundant enhancers, enhancer silencing may be essential to buffer gene expression levels so that they remain within a narrow, biologically tolerable range. On the other hand, small differences in enhancer activities in flies in the wild may serve as a potential source of phenotypic variation that can be acted upon by natural selection. Since population genetic theory predicts that selection differentials of a small fraction of a percent are seen in natural populations with the effective population size of *Drosophila* [20], it is plausible that this variation is functionally significant, perhaps through a subtle influence of haltere morphology on flight performance.

## Materials and Methods

### Genetic variation experiments

The NC2 stocks were obtained from Greg Gibson (N.C. State University); all other wild stocks were obtained from the Bloomington Stock Center (Table S2).

To show that the lack of expression in these outcrosses was not due to a failure to initiate enhancer trap expression in the wild backgrounds, we carried out a lineage tracing experiment. The genotype of the stock was: *Ubx-Gal4^lac1^ UAS-flp; actin>stop>GFP*. The combination of *UAS-flp* and *actin>stop>GFP* records the history (i.e. marks the lineage) of Gal4 expression. When outcrossed to wild backgrounds, *GFP* expression was not silenced (in contrast to when the direct *UAS-GFP* readout was monitored). Together, these results suggest that *Ubx-Gal4^lac1^* was initially activated but then silenced.

Hybrid dysgenesis was ruled out as a reason for loss of expression from P transposons by the following tests: 1) silencing occurs equally well, regardless of the direction the cross was set up, 2) silencing occurs equally well at 18° and 25°C (while hybrid dysgenesis is suppressed at 18°C), 3) silencing was not observed for some other transposon insertions (inside or outside of the *Ubx* locus) when crossed to the same wild stocks, 4) the miniwhite gene associated with the P element insertions did not lead to a variegated eye phenotype as would be expected for somatic transposon excision, and 5) quantitative PCR analysis confirmed that the amount of transposon DNA was the same in the parent (unsilenced) and F2 (silenced) generations. Finally, enhancer trap expression can be recovered when back-crossed into the laboratory stock background.

### Quantification of Ubx protein levels

To measure Ubx protein levels in different genetic backgrounds, we stained haltere discs obtained from uncrowded *yw (2x Ubx+), yw;If/Cyo;TM2/TM6B (1x Ubx+), yw;If/Cyo;DpP5/TM6B (3x Ubx+), yw;DpP10x2/CyoGFP;MKRS/TM6B (4x Ubx+), yw; DpP10x2/CyoGFP;DpP5/DpP5 (6x Ubx+),Hikone-R, Berlin-K, NC2-76, NC2-80, yw x NC2-76 F1s, Tw2, yw x Tw2 F1s, Florida-9, Reids-2, and Harwich* wandering larvae with anti-Ubx (FP3.38) and a fluorescent secondary antibody. Stainings and confocal imaging were done identically and in parallel for ≥8 haltere discs from each genotype. The pixel intensities in identically sized regions of the distal anterior compartments were measured using Adobe Photoshop. This region was quantified because it is a relatively large area that expresses Ubx at uniform levels and gives rise to the main body of the haltere (the same portion measured in Figure 5A and Figure S4A). Similar trends were observed when average pixel intensities for the entire distal haltere were measured. The average intensities for each wild population differed by no more than 16%, suggesting that final Ubx levels are very similar despite differences in genetic background and silencing.

### Quantification of Ubx reporter silencing

To quantify the extent of silencing of the *Ubx-Gal4^lac1^* reporter in response to *Ubx+* copy number and outcrosses to wild populations, third instar haltere discs were dissected from wandering larvae of *yw122; DpP10x2/CyoGFP; Ubx-Gal4^lac1^UAS-GFP/TM6B (4xUbx+)*, and the GFP positive, F1 progeny of *yw122; If/Cyo; Ubx-Gal4^lac1^UAS-GFP/TM6B* crossed with *NC2-80, NC2-76, Ber-2, Tw-2*, and *Harwich*. GFP positive F3 progeny of *yw122; If/Cyo; Ubx-Gal4^lac1^UAS-GFP/TM6B* crossed with NC2-80 and NC2-76 were also dissected. For the outcrosses, we always used females from the wild populations. Haltere discs were fixed, mounted, and imaged for GFP and DAPI on a confocal microscope. Images were made binary in ImageJ. The GFP expressing area relative to the total disc area was measured for each disc, and this value was subtracted from the average GFP expressing area (relative to total disc size) of *yw122; If/Cyo; Ubx-Gal4^lac1^UAS-GFP/TM6B* haltere discs to yield a ‘% silencing’ value for each disc.

### Heat-shock induced Ubx overexpression

Larvae bearing the *hs-UbxIa22* transgene [26] were heat-shocked at 37°C for 15–20 minutes 3 or 4 days after egg laying. Larvae were dissected at least 48 hours after heat shock to allow for total dissipation of exogenous Ubx. *hs-UbxIa22* larvae that were not heat shocked showed no *Ubx* silencing. Neutral clones were induced using the same heat shock regime in flies of the genotype *yw hsflp; FRT 42D Ub-GFP/FRT 42D; hs-UbxIa22/+*.

### *Ubx* enhancer traps and duplications

*Ubx-Gal4^lac1^* [27]; *Ubx-lacZ^lac1^* [28]; *Ubx-Gal4^LDN^* [29]; *Ubx-Gal4^M1^* [29]; *Ubx-lacZ^166^* [30]; and *Ubx-Gal4^M3^* [29]. Although these lines are hypomorphic mutations of the *Ubx* locus, this is unlikely to contribute to our results because decreased production of Ubx would, if anything, cause an underestimate of the amount of silencing that occurs at the Ubx locus.

3x *Ubx+* flies contain a tandem duplication of the *Ubx* locus (Dp(3;3)P5).

4x *Ubx*+ flies contain a tandem duplication of a transpositon of the *Ubx* locus onto the 2^nd^ chromosome (Dp(3;2)P10). Further increases in *Ubx+* copy number were created by combining these duplications [16]. *Ubx^9–22^* expresses a non-functional Ubx protein due to a ∼1500 bp deletion that removes a splice acceptor site and part of the Ubx homeodomain-encoding exon [31].

Before crossing to enhancer traps, *Ubx* duplications were introduced into stocks containing marked chromosomes that do not cause silencing (*yw hsflp; If/cyo; Dp(P5)/Tm6B* and *yw hsflp; Dp(3;2)P10x2/CyoGFP; MKRS/Tm6B*).

To monitor silencing of *Ubx-lacZ^166^* and *Ubx-Gal4^lac1^* simultaneously (Figure S3), flies of the genotype, *Dp(3;2)P10x2/heat shock-Ubx; Ubx-lacZ^166^/Ubx-Gal4^lac1^ UAS-GFP* were given a 15 min. heat shock at 37°C 48 to 96 hrs after egg laying. Imaginal discs were dissected at wandering stage and stained for Ubx, βgal, and GFP. Silencing was not observed in flies of the same genotype without heat shock.

### PcG mutations

*FRT101 ph^504^*

*FRT2A Pc^XT109^*

*FRT42D Su(Z)2^l.b8^*

*FRT82B Scm^D1^*

*FRT42D Pcl^D5^*

Of these mutations, when analyzed in loss-of-function clones, all but *Pcl* resulted in repression of Ubx in the haltere (due to derepression of more posterior Hox genes; data not shown) and therefore could not be used to assess their role in silencing.

### Other lines used

*UAS-GFP Ubx-Gal4^lac1^/TM6B*

*UAS-GFP (X); Ubx-Gal4^LDN^/TM6B*

*UAS-GFP (X); Ubx-Gal4^M1^/TM6B*

*FRT 82B UbxDf(109)/TM6B*

*hs-UbxIa22/TM6B* [26]

*Ubx^9–22^/TM6B*

*vg-Gal4 UAS-GFP*

*vg-Gal4 UAS-GFP UAS-flp act>cd2>Gal4*

*UAS-UbxHA*

*FRT42D Ub-GFP*

*FRT42D Ub-GFP; hs-UbxIa22/Tm6B*

*FRT42D*

*UAS-GFP; FRT42D arm-lacZ; Ubx-Gal4^Lac1^*

*hs-Gal4*

### Antp-Ubx chimeras

(Previously described by [14]

*UAS-Antp*

*UAS-AUA*

UAS-UU* (* refers to a stop codon inserted immediately following the homeodomain)

*UAS-AAU*

*UAS-AUU*

### Quantitative PCR

Whole-fly genomic DNA was isolated from the lab stock containing the *Ubx-Gal4^lac1^* enhancer trap (*yw122; If/CyoGFP; Ubx-Gal4^lac1^ UAS-GFP/TM6B*) and the GFP+ F2 progeny of the *Ubx-Gal4^lac1^* stock crossed to strains Tw2, NC2-76, and NC2-80. Silencing was confirmed to be occurring in these crosses. The F2 progeny were generated by crossing *Gal4^lac1^UAS-GFP* F1 males to wild population females, precluding the possibility of recombination between chromosomes of the lab and wild genotypes. Primers were designed to amplify ∼200 bp in the *Gal4* and *UAS* transgenes to determine their relative abundance in each genotype. A ∼200 bp sequence in the 5′UTR of *homothorax* was amplified to normalize for different amounts of template DNA. PCR amplification was performed in triplicate using Applied Biosystems 7300 Real Time PCR System, and SYBR Green PCR Master Mix. Product dissociation curves were examined to ensure that each primer set only amplified a single product. C_T_ values and amplification curves were consistent with an equal abundance of the *Gal4* and *UAS* sequences in all genotypes.

### Antibody staining

Standard protocols were used with the following primary antibodies:

Rabbit anti-β-Gal 1:10,000 (Cappel)

Mouse anti-En 1:10 (Hybridoma Bank)

Mouse anti-Ubx 1:20

Rat anti-HA 1:100

## Supporting Information

Figure S1 Neutral clones respect the borders of *Ubx* silencing. (A,B) Two examples of haltere discs with neutral clones (marked by the absence of GFP) and *Ubx* silencing (induced by *hs-Ubx*). In (A), there is no crossing between the neutral clones and *Ubx*-silenced patches. In (B), although most of the neutral clones respect the *Ubx*-silenced patches, there are two small exceptions (arrows). *Ubx-* silenced patches are outlined in yellow and the neutral clones are outlined in blue. The exceptions observed in these experiments are likely due to multiple neutral clones that were scored as a single clone because they fused during growth.
(9.78 MB TIF)

Figure S2 PcG functions are required for *Ubx* autoregulatory silencing. (A) Wing disc with *Pcl-* clones (absence of GFP) stained for Ubx (red) and GFP. Ubx expression is observed in pouch clones. (B) Haltere disc with *Pcl-* clones (absence of GFP) stained for Ubx (red) and GFP. Ubx expression is unaffected by the absence of *Pcl*. *Pcl* was the only PcG gene we tested to show strong, autonomous Ubx derepression in the wing disc, and no affect on Ubx expression in the haltere disc; the PcG mutations *Pc, Scm, ph*, and *Su(Z)2* could not be used for this experiment because they result in a loss of *Ubx* expression in the haltere, due to the derepression of more posterior Hox genes in these clones (data not shown). (C) A *Ubx-Gal4^lac1^* haltere disc in which both silencing (by *hs-Ubx*) and *Pcl-* clones were induced. *Pcl-* tissue is outlined in yellow. Silencing of both Ubx and the enhancer trap are observed, but not in *Pcl-* tissue. Note that *Pcl-* clones only affect Ubx expression in the distal, “pouch” domain of the wing and haltere (Beuchle D, Struhl G, Muller J (2001) Polycomb group proteins and heritable silencing of Drosophila Hox genes. Development 128: 993-1004).
(7.73 MB TIF)

Figure S3 Simultaneous monitoring of silencing for two *Ubx* enhancer traps. (A,B) *hs-Ubx*/DpP10x2; UbxGal4^lac1^ UAS-GFP/UbxlacZ^166^ haltere disc from animals that were not given a heat shock (A) or were given a 15 min heat shock (B). The discs were stained for Ubx (blue), GFP (green), and βgal (red). Individual channels are shown as indicated. For (B), where silencing was observed, the outlines of the silenced clones are shown as follows: in the βgal channel (B') the outlines of Ubx (yellow outline) silenced clones are shown. In the GFP channel (B') the outlines of Ubx (yellow outline) silenced clones are shown. B' shows the GFP channel with the *Ubx-lacZ^166^* (red outline) silenced clones. Note that the extent of silencing of *Ubx-Gal4^lac1^* is greater than that of *Ubx-lacZ^166^*, and that *Ubx-lacZ^166^* silencing is a subset of *Ubx-Gal4^lac1^* silencing.
(3.13 MB TIF)

Figure S4 Quantification of haltere sizes and Ubx levels. (A) Quantifications of Ubx protein levels (blue bars) and haltere sizes (red bars) in genotypes with differing numbers of wild type *Ubx+* alleles. Both measurements are shown relative to wild type (2x *Ubx*+). Note that neither measurement scales quantitatively with increases in *Ubx*+ dose, illustrating that these phenotypes are buffered. In contrast, one copy of *Ubx*+ shows a ∼60% reduction in Ubx protein levels and a ∼50% increase in haltere size compared to wild type (2x *Ubx*+). Error bars represent standard error of the mean. (B) Quantifications of Ubx levels in 8 different wild genetic backgrounds (Hikone-R, Berlin-K, NC2-80, NC2-76, Tw2, Florida-9, Reids-2, and Harwich) and two F1s (*yw* X NC2-76 and *yw* X Tw2) are all within ∼16% of those measured in *yw*. Moreover, this variation does not correlate with the degree of silencing (shown in the thumbnail images below the graph). For comparison, halving the dose of *Ubx*+ decreases Ubx levels by ∼40% (left-most bar). Error bars represent standard error of the mean.
(0.36 MB TIF)

Figure S5 *Ubx* silencing increases with introgression into wild genetic backgrounds. (A) *Ubx-Gal4^lac1^* expression in the F1 progeny of a cross to the Tw2 wild type line. (B) Silencing increases when *Ubx-Gal4^lac1^* is introgressed by backcrossing into the Tw2 line. Shown here is a haltere disc after 2 backcrosses (the F3 generation). (C) *Ubx-Gal4^lac1^* expression in the F1 progeny of a cross to the NC2-80 wild type line. (D) Silencing increases when *Ubx-Gal4^lac1^* is introgressed by backcrossing into the NC2-80 line. Shown here is a haltere disc after 2 backcrosses (the F3 generation).
(1.47 MB TIF)

Table S1 Summary of Ubx enhancer traps and their responses to changes in Ubx levels and genetic variation
(0.08 MB DOC)

Table S2 Summary of *Ubx-Gal4^lac1^* silencing in F1 crosses to wild stocks
(0.07 MB DOC)
