# Supplementary figures and images for: Regulation of Ubx Expression by Epigenetic Enhancer Silencing in Response to Ubx Levels and Genetic Variation
Source: PLoS Genet. 2009 Sep 4;5(9):e1000633. doi: 10.1371/journal.pgen.1000633 (PMC2726431; doi:10.1371/journal.pgen.1000633)

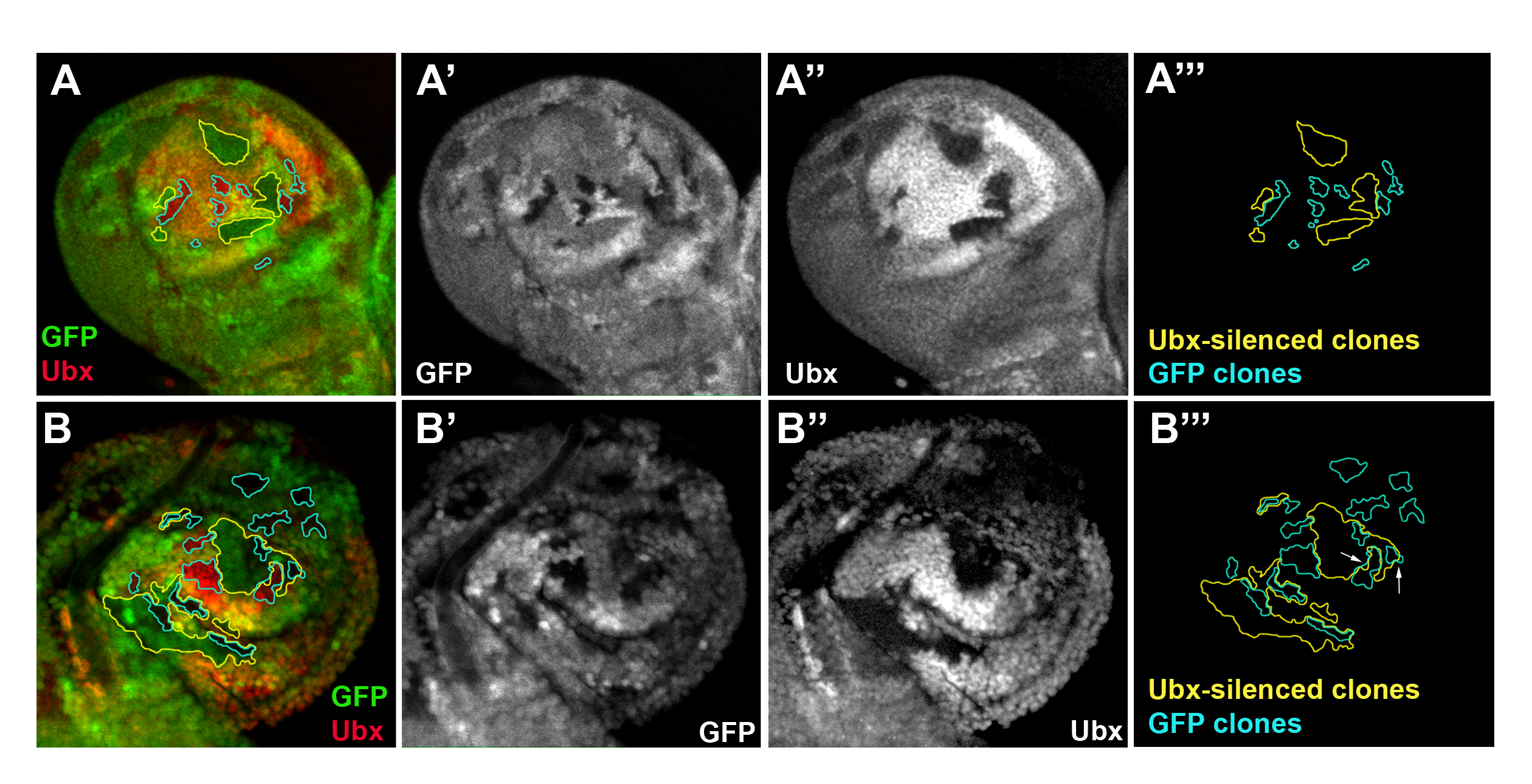

Supplement: Figure S1 — Neutral clones respect the borders of Ubx silencing. (A,B) Two examples of haltere discs with neutral clones (marked by the absence of GFP) and Ubx silencing (induced by hs-Ubx). In (A), there is no crossing between the neutral clones and Ubx-silenced patches. In (B), although most of the neutral clones respect the Ubx-silenced patches, there are two small exceptions (arrows). Ubx- silenced patches are outlined in yellow and the neutral clones are outlined in blue. The exceptions observed in these experiments are likely due to multiple neutral clones that were scored as a single clone because they fused during growth. (9.78 MB TIF) [file pgen.1000633.s001.tif]

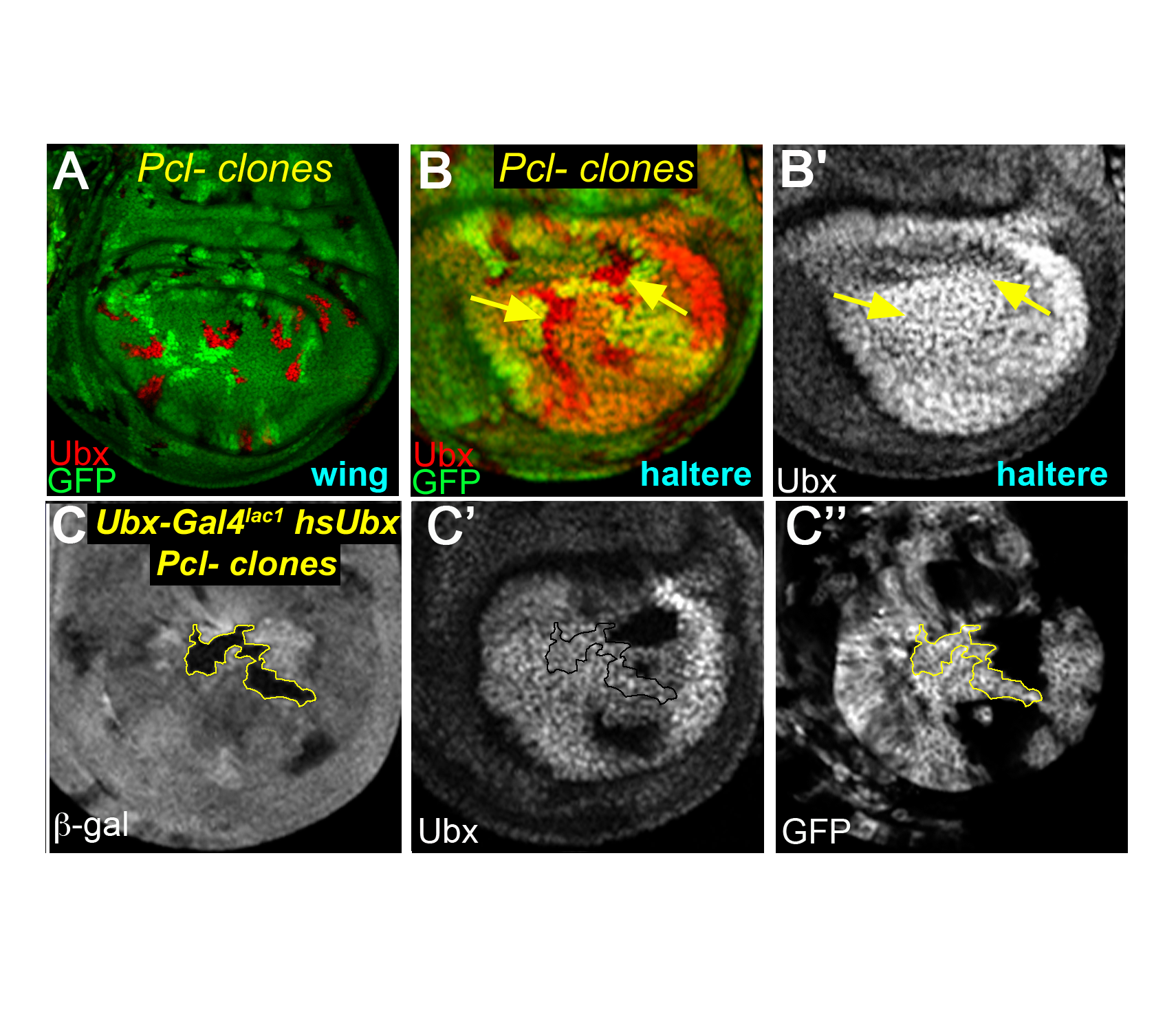

Supplement: Figure S2 — PcG functions are required for Ubx autoregulatory silencing. (A) Wing disc with Pcl- clones (absence of GFP) stained for Ubx (red) and GFP. Ubx expression is observed in pouch clones. (B) Haltere disc with Pcl- clones (absence of GFP) stained for Ubx (red) and GFP. Ubx expression is unaffected by the absence of Pcl. Pcl was the only PcG gene we tested to show strong, autonomous Ubx derepression in the wing disc, and no affect on Ubx expression in the haltere disc; the PcG mutations Pc, Scm, ph, and Su(Z)2 could not be used for this experiment because they result in a loss of Ubx expression in the haltere, due to the derepression of more posterior Hox genes in these clones (data not shown). (C) A Ubx-Gal4lac1 haltere disc in which both silencing (by hs-Ubx) and Pcl- clones were induced. Pcl- tissue is outlined in yellow. Silencing of both Ubx and the enhancer trap are observed, but not in Pcl- tissue. Note that Pcl- clones only affect Ubx expression in the distal, “pouch” domain of the wing and haltere (Beuchle D, Struhl G, Muller J (2001) Polycomb group proteins and heritable silencing of Drosophila Hox genes. Development 128: 993-1004). (7.73 MB TIF) [file pgen.1000633.s002.tif]

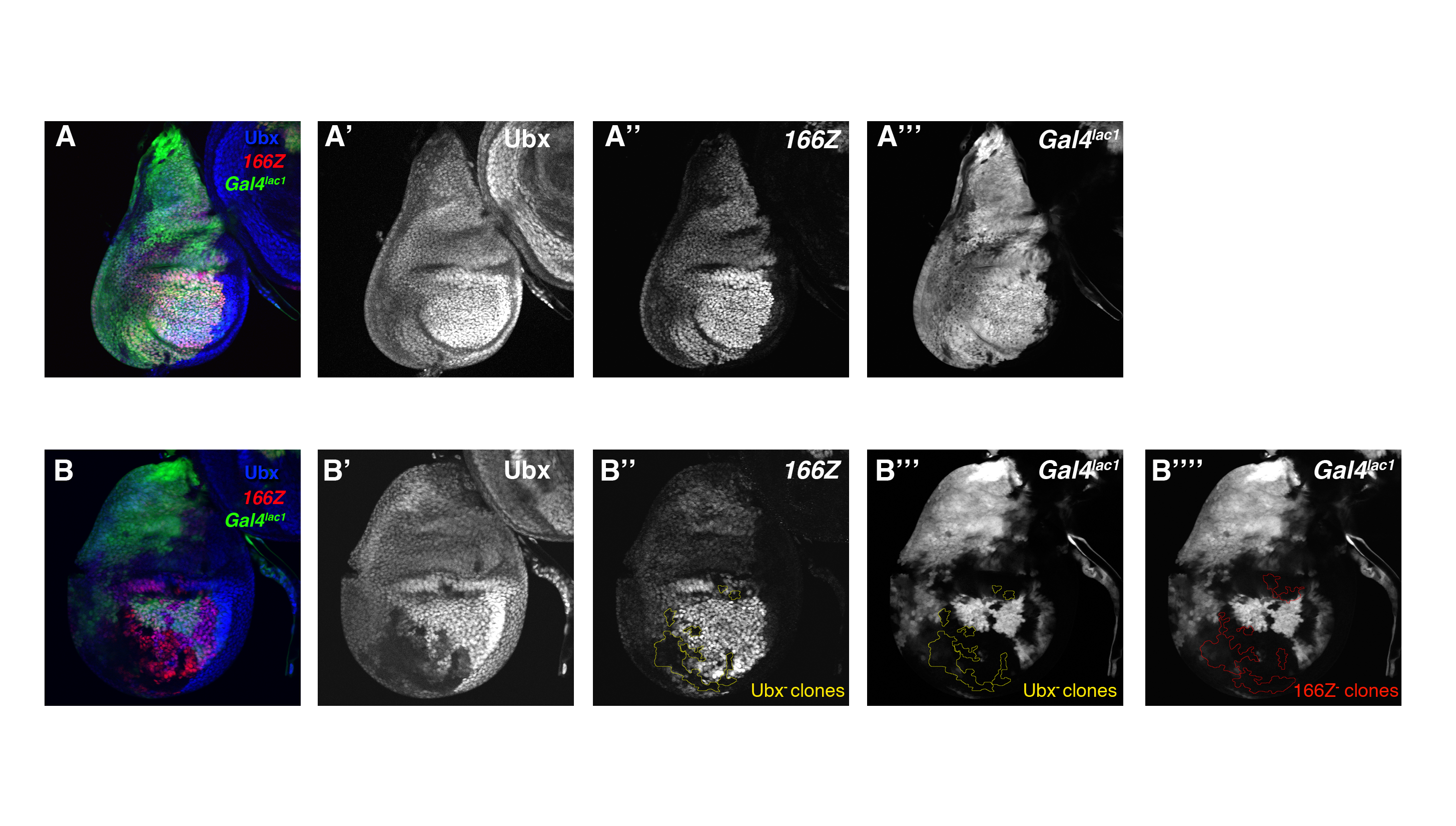

Supplement: Figure S3 — Simultaneous monitoring of silencing for two Ubx enhancer traps. (A,B) hs-Ubx/DpP10x2; UbxGal4lac1 UAS-GFP/UbxlacZ166 haltere disc from animals that were not given a heat shock (A) or were given a 15 min heat shock (B). The discs were stained for Ubx (blue), GFP (green), and βgal (red). Individual channels are shown as indicated. For (B), where silencing was observed, the outlines of the silenced clones are shown as follows: in the βgal channel (B') the outlines of Ubx (yellow outline) silenced clones are shown. In the GFP channel (B') the outlines of Ubx (yellow outline) silenced clones are shown. B' shows the GFP channel with the Ubx-lacZ166 (red outline) silenced clones. Note that the extent of silencing of Ubx-Gal4lac1 is greater than that of Ubx-lacZ166, and that Ubx-lacZ166 silencing is a subset of Ubx-Gal4lac1 silencing. (3.13 MB TIF) [file pgen.1000633.s003.tif]

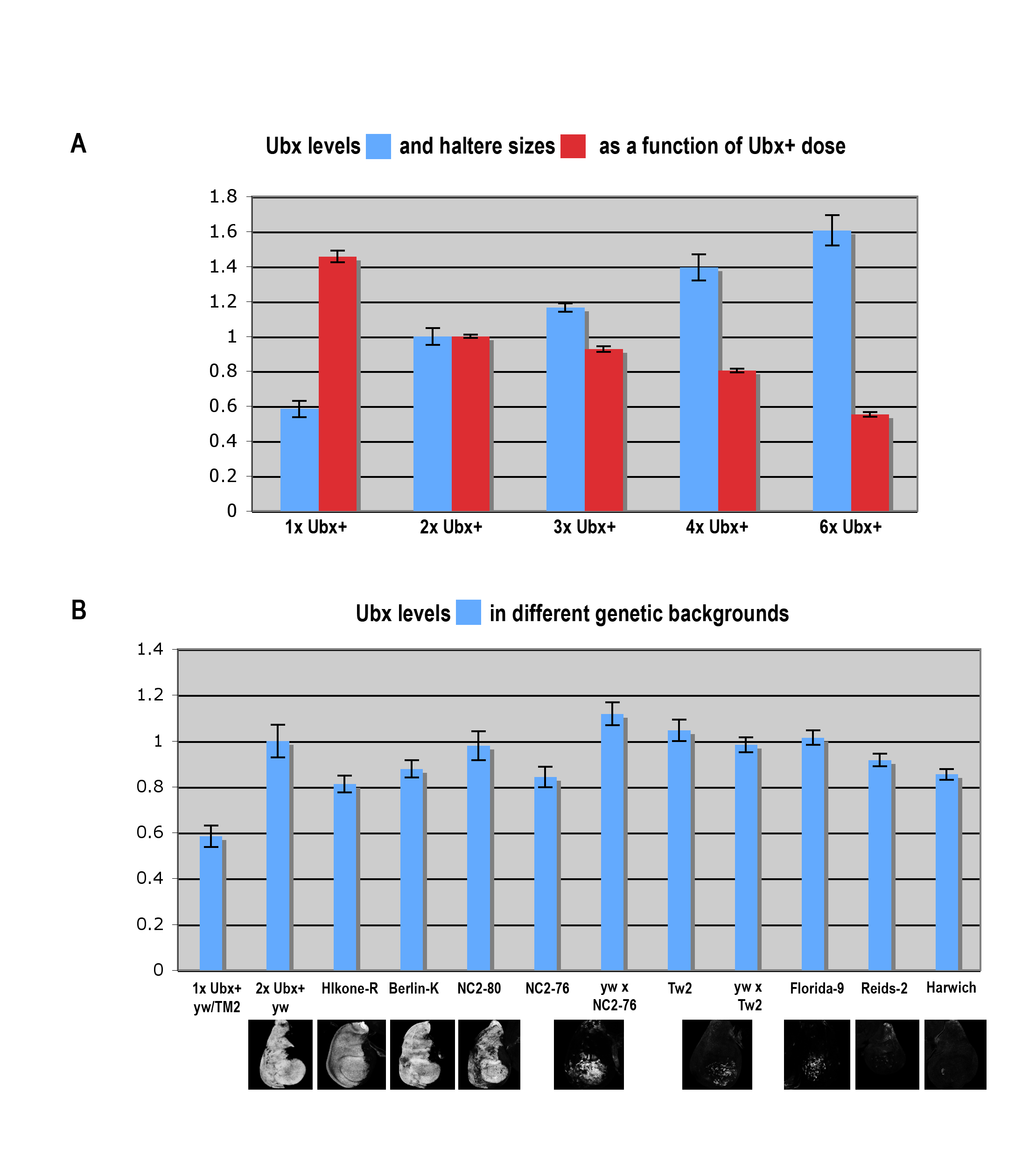

Supplement: Figure S4 — Quantification of haltere sizes and Ubx levels. (A) Quantifications of Ubx protein levels (blue bars) and haltere sizes (red bars) in genotypes with differing numbers of wild type Ubx+ alleles. Both measurements are shown relative to wild type (2x Ubx+). Note that neither measurement scales quantitatively with increases in Ubx+ dose, illustrating that these phenotypes are buffered. In contrast, one copy of Ubx+ shows a ∼60% reduction in Ubx protein levels and a ∼50% increase in haltere size compared to wild type (2x Ubx+). Error bars represent standard error of the mean. (B) Quantifications of Ubx levels in 8 different wild genetic backgrounds (Hikone-R, Berlin-K, NC2-80, NC2-76, Tw2, Florida-9, Reids-2, and Harwich) and two F1s (yw X NC2-76 and yw X Tw2) are all within ∼16% of those measured in yw. Moreover, this variation does not correlate with the degree of silencing (shown in the thumbnail images below the graph). For comparison, halving the dose of Ubx+ decreases Ubx levels by ∼40% (left-most bar). Error bars represent standard error of the mean. (0.36 MB TIF) [file pgen.1000633.s004.tif]

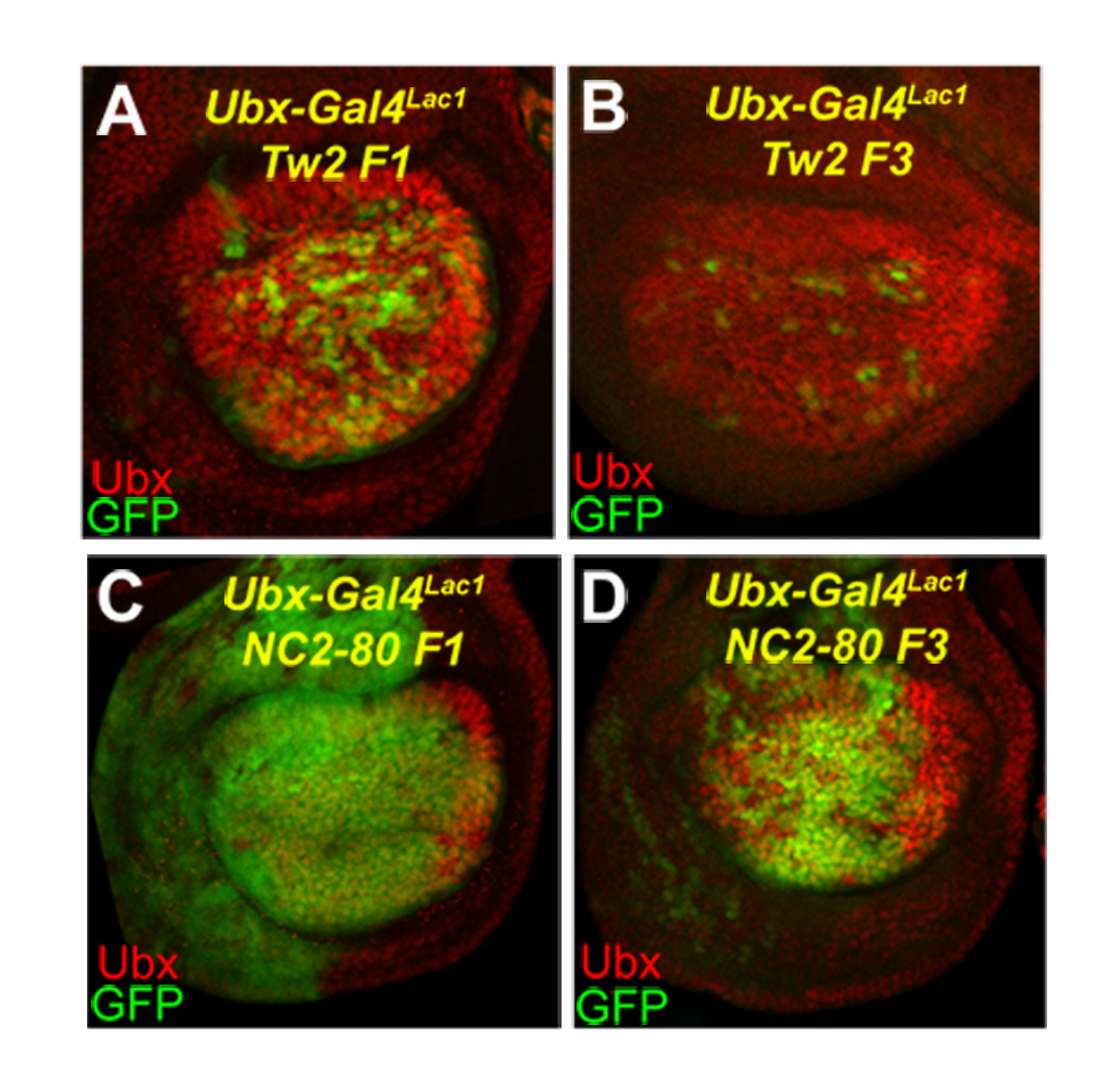

Supplement: Figure S5 — Ubx silencing increases with introgression into wild genetic backgrounds. (A) Ubx-Gal4lac1 expression in the F1 progeny of a cross to the Tw2 wild type line. (B) Silencing increases when Ubx-Gal4lac1 is introgressed by backcrossing into the Tw2 line. Shown here is a haltere disc after 2 backcrosses (the F3 generation). (C) Ubx-Gal4lac1 expression in the F1 progeny of a cross to the NC2-80 wild type line. (D) Silencing increases when Ubx-Gal4lac1 is introgressed by backcrossing into the NC2-80 line. Shown here is a haltere disc after 2 backcrosses (the F3 generation). (1.47 MB TIF) [file pgen.1000633.s005.tif]
