# Supplementary material for: Regulation of Ubx Expression by Epigenetic Enhancer Silencing in Response to Ubx Levels and Genetic Variation
Source: PLoS Genet. 2009 Sep 4;5(9):e1000633. doi: 10.1371/journal.pgen.1000633 (PMC2726431; doi:10.1371/journal.pgen.1000633)
Supplement: Table S1 — Summary of Ubx enhancer traps and their responses to changes in Ubx levels and genetic variation (0.08 MB DOC) [file pgen.1000633.s006.doc]

**Table S1.** Summary of Ubx enhancer traps and their responses to changes in Ubx levels and genetic variation.

| ***Ubx* readout** | **reference** | **Wild type pattern** | ***hs-Ubx*** | **4x *Ubx+*** | **X Tw2 or NC2-76** |
| --- | --- | --- | --- | --- | --- |
| Ubx | this work; White and Wilcox, 1984 | Throughout the haltere, higher distally, higher in P | Clones | No change | No change |
| *Gal4^lac1^* | [1] | All of A, distal P | Clones | Clones | Clones |
| *lacZ^lac1^* | [2] | All of A (stronger distal), weak distal P | ND | Generally weaker; off proximal | Same as 4x *Ubx+* |
| *Gal4^M1^* | [3] | A+P, higher distal | Clones | Generally weaker; off proximal | Same as 4x *Ubx+* |
| *Gal4^M3^* | [3] | Distal (A patchy, P strong) | ND | No change | No change |
| *Gal4^LDN^* | [3] | A+P, distal only | Clones | Clones | Clones |
| *lacZ^166^* | [4] | Accurate in A, off in P | Clones | Generally weaker | Same as 4x *Ubx*+ |

A, anterior compartment; P, posterior compartment. "Clones" means the insertion was silenced in groups of haltere cells. ND, not determined

References:

1. Pallavi SK, Shashidhara LS (2003) Egfr/Ras pathway mediates interactions between peripodial and disc proper cells in Drosophila wing discs. Development 130: 4931-4941.

2. Casares F, Bender W, Merriam J, Sanchez-Herrero E (1997) Interactions of Drosophila Ultrabithorax regulatory regions with native and foreign promoters. Genetics 145: 123-137.

3. de Navas L, Foronda D, Suzanne M, Sanchez-Herrero E (2006) A simple and efficient method to identify replacements of P-lacZ by P-Gal4 lines allows obtaining Gal4 insertions in the bithorax complex of Drosophila. Mech Dev 123: 860-867.

4. Bender W, Hudson A (2000) P element homing to the Drosophila bithorax complex. Development 127: 3981-3992.
