# Supplementary material for: Regulation of Ubx Expression by Epigenetic Enhancer Silencing in Response to Ubx Levels and Genetic Variation
Source: PLoS Genet. 2009 Sep 4;5(9):e1000633. doi: 10.1371/journal.pgen.1000633 (PMC2726431; doi:10.1371/journal.pgen.1000633)
Supplement: Table S2 — Summary of Ubx-Gal4lac1 silencing in F1 crosses to wild stocks (0.07 MB DOC) [file pgen.1000633.s007.doc]

**Table S2. Summary of *Ubx-Gal4^lac1^* silencing in F1 crosses to wild stocks.**

| **Name** | **Silencing of *Ubx-Gal4^lac1^*** | **Provenance** | **Immediate Source** | **Stock #** |
| --- | --- | --- | --- | --- |
| BER 1 | None | Bermuda, 1954 | Bloomington Stock Center | 3839 |
| Berlin K | None | - | Bloomington Stock Center | 8522 |
| BOG 1 | None | Bogota, Colombia, 1962 | Bloomington Stock Center | 3841 |
| Canton-S | None | - | Bloomington Stock Center | 1 |
| Crimea | None | - | Bloomington Stock Center | 4266 |
| Hikone A-W | None | - | Bloomington Stock Center | 4 |
| Hikone R | None | - | Bloomington Stock Center | 4267 |
| KSA 2 | None | Koriba Dam, South Africa, 1963 | Bloomington Stock Center | 3852 |
| NO 1 | None | New Orleans, USA, 1954 | Bloomington Stock Center | 3860 |
| Oregon-R | None | - | Leslie Voshall |  |
| PVM | None | Madeira, Portugal, 1965 | Bloomington Stock Center | 3861 |
| Qi 2 | None | Israel, 1954 | Bloomington Stock Center | 3864 |
| Samarkand | None | - | Bloomington Stock Center | 4270 |
| Vag 2 | None | Athens, Greece, 1965 | Bloomington Stock Center | 3876 |
| KSA 3 | Weak | Koriba Dam, South Africa, 1963 | Bloomington Stock Center | 3853 |
| Amherst-3 | Weak | - | Bloomington Stock Center | 4265 |
| NC2-64 | Weak | North Carolina, USA, 2000 | Greg Gibson |  |
| NC2-80 | Weak | North Carolina, USA, 2000 | Greg Gibson |  |
| NC2-96 | Weak | North Carolina, USA, 2000 | Greg Gibson |  |
| PYR-2 | Weak | Pyrenees, Spain, 1965 | Bloomington Stock Center | 3862 |
| BER 2 | Strong | Bermuda, 1954 | Bloomington Stock Center | 3840 |
| BOG 2 | Strong to moderate | Bogota, Colombia, 1962 | Bloomington Stock Center | 3842 |
| CO 3 | Strong to moderate | Commack, New York, USA 1961 | Bloomington Stock Center | 3848 |
| Florida 9 | Strong to moderate | - | Bloomington Stock Center | 2374 |
| Harwich | Strong | - | Bloomington Stock Center | 4264 |
| NC2-27 | Strong | North Carolina, USA, 2000 | Greg Gibson |  |
| NC2-54 | Strong | North Carolina, USA, 2000 | Greg Gibson |  |
| NC2-76 | Strong | North Carolina, USA, 2000 | Greg Gibson |  |
| NC2-9 | Strong | North Carolina, USA, 2000 | Greg Gibson |  |
| NC2-91 | Strong | North Carolina, USA, 2000 | Greg Gibson |  |
| Reids 2 | Strong | Madeira, Portugal, 1965 | Bloomington Stock Center | 3867 |
| TW2 | Strong | Varna, New York, USA 1977 | Bloomington Stock Center | 3873 |
|  |  |  |  |  |
|  |  |  |  |  |
|  |  |  |  |  |
|  | **Tested:** | **32** |  |  |
|  | **No silencing:** | **14** |  |  |
|  | **Weak:** | **6** |  |  |
|  | **intermediate - Strong:** | **12** |  |  |
